# Characterization of glyphosate-tolerant genetically modified eucalyptus

**DOI:** 10.1080/21645698.2024.2429200

**Published:** 2024-11-24

**Authors:** Antonio Carlos Mota Porto, José Mateus Wisniewski Gonsalves, Paula Aparecida Vieira, Matheus Perek, Diego da Costa Lima, Marcio Nagayschi, Thais Regina Drezza, Ana Cristina Pinheiro, Eduardo Jose de Mello, Dror Avisar, Rodrigo Neves Graca

**Affiliations:** a Suzano S.A. (FuturaGene - Biotech Division), Itapetininga, Brazil; b FuturaGene Israel Ltd. (R&D), Rehovot, Israel

**Keywords:** CP4-EPSPS, GM eucalyptus, herbicide-tolerance, photosynthesis, weed control

## Abstract

Eucalyptus stands out as one of the most productive tree species for large-scale cultivation. However, like all cultivated crops, it requires specialized management practices, including the control of weeds, pathogens, and pests. Glyphosate is the most widely applied herbicide used in the essential weeding effort, and it ensures the sustainable management of eucalyptus cultivation in Brazil. Given the sensitivity of eucalyptus to glyphosate, existing weed control methods in young eucalyptus farms predominantly rely on protected mechanical or/and knapsack spraying. Both methods contribute to herbicide drift, which compromises tree yield and increases chemical waste due to uneven spraying. This study provides a detailed observation of the physiological parameters and long-term field performance of glyphosate-tolerant (HT), genetically modified (GM) eucalyptus developed by FuturaGene/Suzano S.A. and approved in Brazil for operational deployment. The HT GM eucalyptus events were meticulously evaluated to ensure high levels of glyphosate tolerance. This involved the direct application of herbicide on seedlings in greenhouse studies and on young trees in field conditions. The herbicide-treated GM eucalyptus in all trials demonstrated consistent growth and maintained physiological parameters comparable to their respective non-sprayed wild-type (WT) counterparts. The HT GM eucalyptus represents a significant advancement by enabling the direct application of glyphosate over the top of the trees to control the weeds within the planting row. This innovative approach minimizes the need for frequent mechanical and manual interventions, thereby lowering worker herbicide exposure, reducing the environmental impact of mechanical operations, and enhancing the overall efficiency and sustainability of HT GM eucalyptus stands.

## Introduction

Eucalyptus species have great potential to contribute to the growing demand of woody products due to their rapid growth rates, adaptability to various environmental conditions, and economic value in diverse industries.^1–3^ However, in recent years, eucalyptus cultivation has faced challenges due to biotic and abiotic stresses (e.g., high temperatures, water scarcity, pests, pathogens, weed competition, etc.), which reduce their productive potential and increase operational costs.^1,4–6^ Weeds can significantly affect the survival and growth of eucalyptus trees by competing for light, water, and nutrients.^7–10^ Their proportions can vary by site, planting density, and eucalyptus genotypes.^9,11–14^ Weed competition can lead to substantial reductions in the initial growth of eucalyptus, attenuating wood volume by 55–87%.^15,16^ The long-term growth response can be even more affected, with a 61–91% reduction in wood volume.^13,17^ Therefore, sustainable management of eucalyptus farms requires effective weed control strategies to mitigate competition for resources and maximize yields.

Chemical weed control methods, coupled with other management techniques such as spacing optimization, are commonly employed worldwide.^18,19^ Herbicide application is the most common method to control weed competition in eucalyptus, with glyphosate being the most widely used reagent in Brazil. It is typically applied across the entire area before planting during site preparation, between the rows after planting, and following harvesting to manage coppice regrowth by effectively eliminating remaining stumps.^20–24^

Eucalyptus is highly sensitive to glyphosate, with productivity losses measured even after exposure to low doses.^24–28^ Despite using protective barriers during glyphosate application to safeguard eucalyptus seedlings, recent studies suggest its inefficacy and tendency to generate drift.^29^ Drift of glyphosate subdoses (<16%) on Eucalyptus can cause severe phytotoxicity, reducing photosynthetic activity, leading to plant death and reducing biomass by up to 34% in the first year of growth.^25,30^ Furthermore, this approach is labor-intensive, costly, and carries a high risk of damage to young seedlings.^31–33^

Current traditional weed management approaches in commercial eucalyptus stands involve protected mechanical spraying to control the weeds between the planted rows, requiring the tractor to pass between all planted rows in the farm.^17,21,34^ This is followed by extensive manual activities to control the weeds within the planted rows, including herbicide application with backpack sprayers and mechanical weeding with hoes.^35^ These labor-intensive practices pose challenges in terms of cost, weed-control effectiveness, herbicide drift, and environmental impact due to wasted fossil fuels and soil compaction from frequent tractor passes.^36^ In this context, developing eucalyptus with glyphosate tolerance presents a promising solution to address these challenges.

The first and most obvious advantage is the protection of yield from glyphosate drifts. It allows over-the-top spraying, which controls the weeds between and inside the planted rows in 6–8 rows with one tractor pass. This significantly reduces the environmental footprint of mechanical operations and carbon usage, reduces the need for manual labor, and enhances worker health and well-being. Implementing uniform over-the-top spraying throughout the farm reduces chemical use, controls the weeds more efficiently, and protects the yield, ensuring the economic viability of eucalyptus cultivation.

Given the absence of tolerance variability across eucalyptus breeding populations, FuturaGene/Suzano has pioneered the development of genetically modified (GM) glyphosate-tolerant eucalyptus varieties. These GM eucalyptus events carry the gene epsps from *Agrobacterium tumefaciens* strain cp4, which encodes a glyphosate-tolerant 5-enolpyruvyl-shikimate-3-phosphate synthase (EPSPS) enzyme.^24,37^ This homologue has a low affinity for glyphosate but a high affinity for phosphoenolpyruvate (PEP), thereby serving as a bypass to the endogenous EPSPS whenever glyphosate is applied.^24,37–41^

The primary goal of this research is to evaluate the glyphosate-tolerant GM eucalyptus events 751K022, 955S019, and 955S024 under controlled greenhouse and field conditions. By comparing the tolerance levels of two wild-type (WT) clones and three GM events, the study aims to determine the effects of glyphosate application on plant phytotoxicity, biomass, and photosynthesis parameters. The research also seeks to assess long-term growth performance, including height, diameter, and tree volume, following herbicide treatment. Statistical analyses, such as analysis of variance and Tukey tests, are employed to ensure reliable comparisons across different genotypes and treatment levels.

This work details the development and selection of the glyphosate-tolerant GM eucalyptus events 751K022, 955S019, and 955S024. Comprehensive assessments of growth rate and photosynthetic parameters meticulously guided the selection and deployment of these events. This comprehensive evaluation aims to provide insights into the efficacy of glyphosate-tolerant eucalyptus events and their potential for improved weed management in forestry practices.

## Materials and Methods

### Early Evaluation of Glyphosate Tolerance Under Greenhouse Conditions

The experiment was carried out in a polycarbonate greenhouse equipped with a cooling system-type pad-fan, located in Itapetininga, Sao Paulo, Brazil. The trials were conducted between May and June of 2023, with internal temperatures ranging between 18°C and 28°C, and the relative humidity ranging between 70% and 90%. Two WT *Eucalyptus ssp*. hybrid clones and three glyphosate-tolerant GM events (Table 1), derived from the WT clones, were evaluated for their tolerance to direct application of increasing doses of the herbicide glyphosate, commercial product Scout^Ⓡ^ (0, 0.45, 1.8 or 3.6 kg a. e. ha^−1^).

**Table 1.** *Eucalyptus urophylla* genotypes tested in the experiment.

#	Genotype	Background	Type	Species
1	751K022	FGN-K	GM Event	*Eucalyptus urophylla*
2	955S019	FGN-S	GM Event	*Eucalyptus urophylla*
3	955S024	FGN-S	GM Event	*Eucalyptus urophylla*
4	FGN-K	FGN-K	Wild type	*Eucalyptus urophylla*
5	FGN-S	FGN-S	Wild type	*Eucalyptus urophylla*

Plants (120 days old) were transplanted individually into 1.2 L pots filled with organic medium (sphagnum peat, expanded vermiculite, dolomitic limestone, agricultural gypsum and NPK fertilizer). Water and nutrients were provided by a drip system. Glyphosate herbicide was applied, at different doses of the acid equivalent, inside of an herbicide-spray booth, using an automated spray-bar equipped with a model TeeJet XR nozzle with a flat-fan pattern and 110-degree spray angle. The system was calibrated to deliver the equivalent of 200 L ha^−1^.

**Figure 1.**
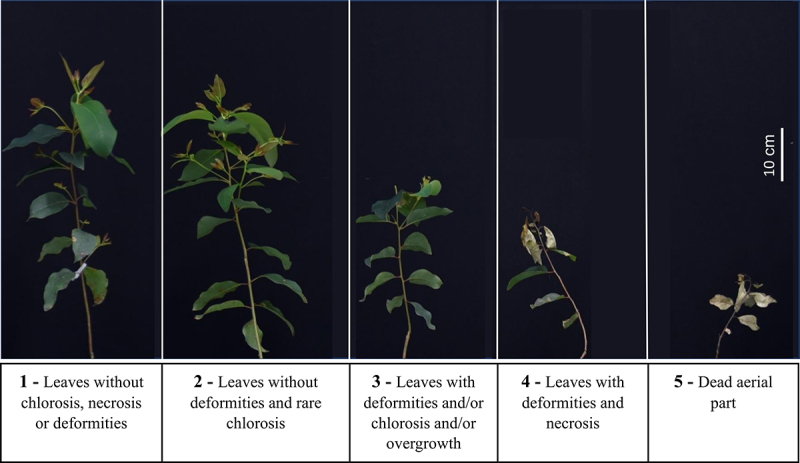
Phytotoxicity scale.

The experiment was designed in completely randomized with four replications (one-plant-plot) with a double factorial 5 (Dose) x 5 (Genotype) design. The herbicide was sprayed 7 days after transplanting. Evaluations were carried out 15 days after application (DAA) and included visual assessment of phytotoxicity symptoms (Figure 1) and weighing the aerial part biomass (g).

Analysis of variance was conducted within genotypes and among doses. Means separations were determined by the Tukey test, at a 5% error probability.

### Evaluation of Photosynthesis Parameters Under Greenhouse Conditions

The experiment was set up in a polycarbonate greenhouse equipped with a cooling system-type pad-fan, located in Itapetininga, Sao Paulo, Brazil. The trials were conducted between May and June of 2023, the internal temperatures ranged between 18°C and 28°C, and the relative humidity was between 70% and 90%. Plants (120 days old) were transplanted individually into 18 L pots filled with organic medium (sphagnum peat, expanded vermiculite, dolomitic limestone, agricultural gypsum and NPK). Water and nutrients were provided by a drip system. The treatments were arranged in a double factorial scheme of 2 (treatments) x 5 (genotypes). More specifically, all five genotypes were evaluated at two treatments levels: (*i*) control, where no herbicide was applied; (*ii*) sprayed, where the potted plant was sprayed with 1.8 kg of acid equivalent of glyphosate per hectare (1.8 kg a.e. ha^−1^) using the commercial product Scout^Ⓡ^ 30 days after transplanting. Each treatment included three plants of each event and three seedlings of the non-transformed conventional clone (WT).

All plants were evaluated one day before herbicide application and daily for 5 days after herbicide application. Photosynthesis parameters were assessed with LI-6800 (LI-COR, Lincoln, NE, USA), an apparatus that measures gas exchange and leaf fluorescence. The level of photosynthesis was measured under 10 different light intensities (activation of photosynthetic radiation), ranging from zero to 2600 μmol photons m^−2^ s^−1 40,41^. The light response curves were used to determine the light compensation point (LSP), maximum net photosynthetic rate (A_max_) and apparent quantum yield (AQY).^42,43^

The data were subjected to analysis of variance and the means of LSP, Amax and AQY were compared between genotypes, within each treatment and after herbicide application were tested by the Tukey test, at 5% probability of error.

### Long-Term Yield Evaluation in Field Trials

Two event-selection field trials were conducted in different seasons and sites to compare the long-term development of the events and respective WTs after early application of glyphosate (Table 2). The trials were conducted in randomized blocks with herbicide treatment in strips. Five replications of linear plots of two plants were used.

**Table 2.** Sites characteristics and planting conditions of long-term field trials.

Information	Site 1	Site 2
Site code, location	Sao Paulo State, Brazil	Sao Paulo State, Brazil
Spacing	3 m x 2.5 m	3 m × 2 m
Elevation (m)	709 m	627 m
Soil taxonomy	Entisol	Oxisol
Climate^a^	Cwa	Cfa
Precipitation	1352	1324
Average temperature	22.0	20.7
Planting date	Dec-19	Oct-18
Evaluation date	May-21	Sep-21

^a^Köppen climate classification.

For control plots, there was no herbicide application and weed control was done with mechanical weeding. Treated plots were sprayed with glyphosate herbicide (1.8 kg a.e. ha^−1^) 30 and 60 days after planting. The WT clones were protected from herbicide drift with the help of a plastic tarpaulin. Total height and diameter at breast height (1.3 m) were collected and the volume of each tree was calculated (Table 2). Linear mixed models were used to extract the best linear unbiased predictors (BLUPs) of the genotypes within treatment which were compared by their standard error of prediction (SEP).^44^

## Results

### Early Evaluation of Glyphosate Tolerance Under Greenhouse Conditions

**Figure 2.**
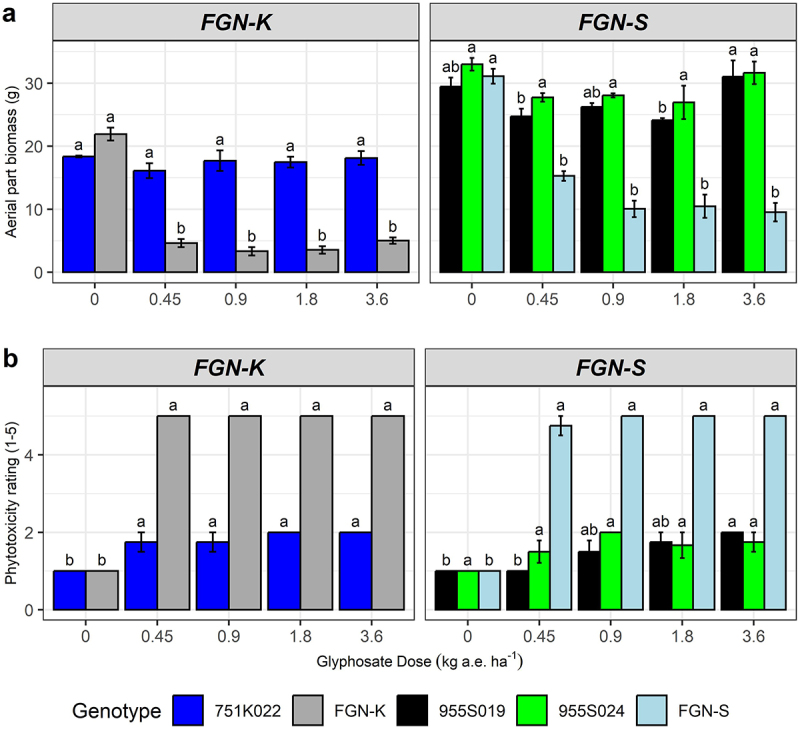
Response of glyphosate-tolerant GM eucalyptus 751K022, 955S019 and, 955S024 and their wt-backgrounds fgn-K and FGN-S under 0, 25%, 50%, 100% and, 200% of acid equivalent ha^−1^ of glyphosate using the commercial product Scout® at 15 days after application (DAA). (a) aerial part biomass (g), (b) phytotoxicity rating (1-5). Means followed by the same letter do not differ statistically by the Tukey test at 5% significance level.

WT seedlings treated with different doses of glyphosate showed impaired development and maintenance of the growth development (*p-value* < 0.05) (Figure 2). No statistical reduction was noted in the aerial part biomass of the three events at the highest dose of glyphosate (3.6 kg a.e. ha^−1^). At the lowest tested dose (0.45 kg a.e. ha^−1^) a drastic reduction in the aerial part biomass of the WT genotypes was observed, with a reduction of 78% for the FGN-K genotype and 51% for the FGN-S genotype (Figure 2a).

The events 751K032 and 955S024 had statistically the same performance at all glyphosate doses, while the event 955S019 had a slight statistically significant decrease in biomass at doses of 0.45, 0.9 and 1.8 kg a.e. ha^−1^ comparing to the control treatment, although the highest aerial part biomass was observed at the maximum dose tested (3.6 kg a.e. ha^−1^) (Figure 2a).

At the lowest tested dose (0.45 kg a.e. ha^−1^), glyphosate triggered phytotoxic symptoms in the WT clones, which culminated in the death of the aerial part (score = 5). In contrast, phytotoxicity scores between 1 and 2 were documents for events 955S019 and 955S024 after treatment with the different glyphosate doses. The average phytotoxicity score for event 751K022 was 2 in the two treatments with higher doses of glyphosate 1.8 and 3.6 kg a.e. ha^−1^. In general, the events presented leaves without chlorosis, necrosis or deformities or evolved into symptoms of rare chlorosis in the leaves (Figure 2b).

### Evaluation of Photosynthesis Parameters Under Greenhouse Conditions

**Figure 3.**
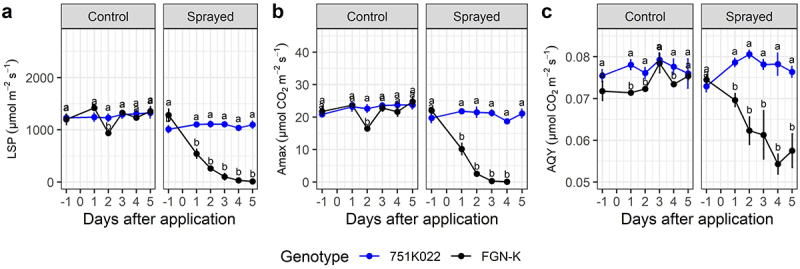
Photosynthetic parameters of GM Eucalyptus glyphosate-tolerant 751K022 and their wt-background FGN-K under the recommended dose of glyphosate (1.8 kg acid equivalent ha^−1^ of glyphosate using the commercial product Scout®) applied 30 days post-planting and evaluated up to 5 days post-application. (a) Light compensation point (LSP), (b) maximum net photosynthetic rate (A_max_), and (c) apparent quantum yield (AQY). Means followed by the same letter do not differ statistically between genotypes, within each treatment, by the Tukey test at 5% significance level.

**Figure 4.**
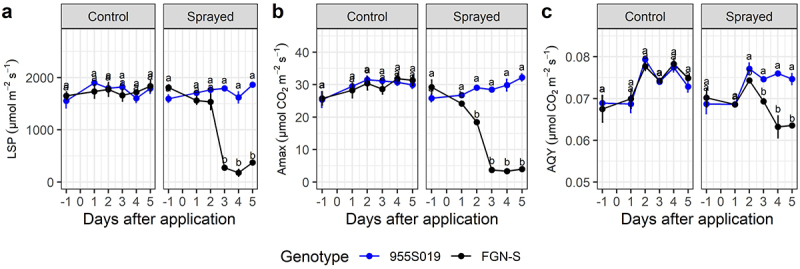
Photosynthetic parameters of GM Eucalyptus glyphosate-tolerant 955S019 and its wt-background FGN-S under the recommended dose of glyphosate (1.8 kg acid equivalent ha^−1^ of glyphosate using the commercial product Scout®) applied 30 days post-planting and evaluated up to 5 days post-application. (a) Light compensation point (LSP), (b) maximum net photosynthetic rate (A_max_), and (c) apparent quantum yield (AQY). Means followed by the same letter do not differ statistically between genotypes, within each treatment, by the Tukey test at 5% significance level.

**Figure 5.**
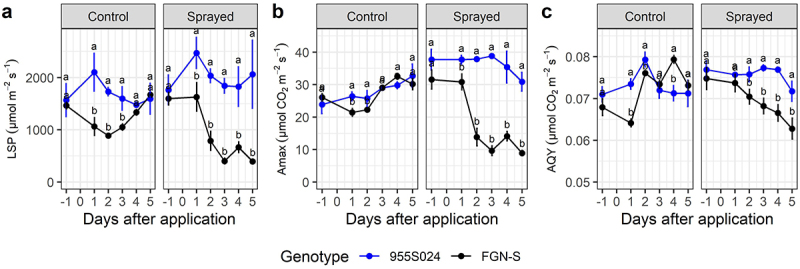
Photosynthetic parameters of GM Eucalyptus glyphosate-tolerant 955S024 and their wt-background FGN-S under the recommended dose of glyphosate (1.8 kg acid equivalent ha^−1^ of glyphosate using the commercial product Scout®) applied 30 days post-planting and evaluated up to 5 days post-application. (a) Light compensation point (LSP), (b) maximum net photosynthetic rate (A_max_), and (c) apparent quantum yield (AQY). Means followed by the same letter do not differ statistically between genotypes, within each treatment, by the Tukey test at 5% significance level.

Following glyphosate application, WT exhibited statistically significantly reduced photosynthetic measures as compared to the glyphosate-tolerant events (*p-value* < 0.05) (Figures 3–5). In general, all events demonstrated the same photosynthetic behavior both before and after glyphosate application, which was similar to that of the pre-treatment WT clones (Figures 3–5). A comparison of event 751K022 with its respective WT FGN-K found a strong reduction in LSP on 1 DAA (Figure 3a). The same pattern was observed for maximum net photosynthetic rate (A_max_) and AQY, showing that WT is more sensitive to glyphosate, and suffers from a drop in photosynthetic efficiency shortly after glyphosate application (Figure 3).

In general, LSP (µmol m^−2^ m^−1^) and A_max_ (µmol CO_2_ m^−2^ m^−1^) better discriminated between the WT clones and the events following glyphosate treatment. For example, at 5 DAA, the gradient of decreasing parameter values between events and WTs was 81–91% for LSP, 71–99% for Amax, and 13.8–25% for AQY (Figures 3–5). Events from the FGN-S background had less drastic responses than events from the FGN-K background but were still very noticeable when maintaining the photosynthetic efficiency of events 955S019 and 955S024 when compared under control treatment and sprayed treatment (Figures 4 and 5).

A decrease in the AQY began at 3 DAA and measured 0.069 µmol CO_2_ m^−2^ m^−1^ was observed in the event 955S019 versus 0.074 µmol CO_2_ m^−2^ m^−1^ in the WT FGN-S (Figure 4c). At 5 DAA (0.063 µmol CO_2_ m^−2^ m^−1^), event 955S019 presented an average AQY of 0.074 µmol CO_2_ m^−2^ m^−1^, which was 15% higher compared to the WT treated with the same dose.

At 3 DAA, event 955S024 had an A_max_ of 38.8 µmol CO_2_ m^−2^ m^−1^, while the FGN-S WT had an A_max_ of 9.62 µmol CO_2_ m^−2^ m^−1^, representing a 75% reduction compared to the event treated with glyphosate (Figure 5b). At 5 DAA, the treated WT (8.6 µmol CO_2_ m^−2^ m^−1^) exhibited an average 72% lower A_max_ compared to the WT under control treatment and event 955S024 under control and sprayed treatment (31.2 µmol CO_2_ m^−2^ m^−1^) (Figure 5b).

### Long-Term Yield Evaluation in Field Trials

**Figure 6.**
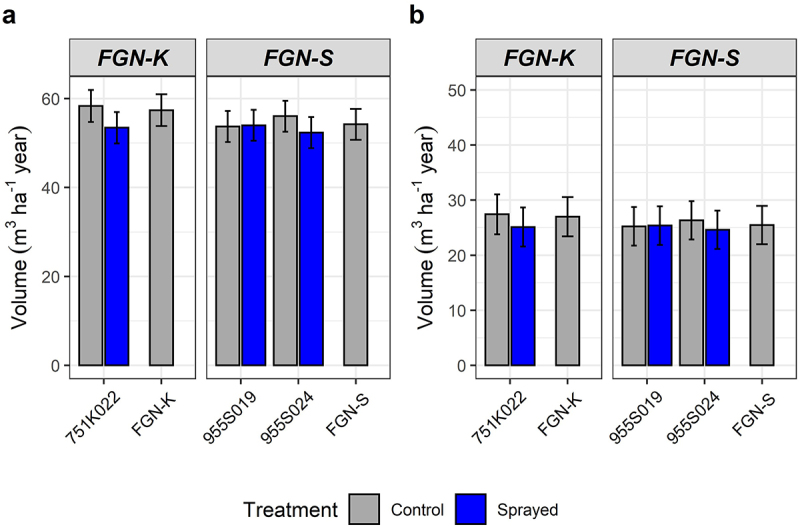
Yield performance (volume – m^3^ ha^−1^ year) of glyphosate-tolerant GM eucalyptus and their backgrounds (WT) after two over-the-top applications of glyphosate at 30- and 60-days after planting. (a) field trial 1, roundwood volume evaluated 17 months after planting. (b) field trial 2, roundwood volume evaluated 36 months after planting.

Long-term field evaluations showed that events and WT yielded similar roundwood volume (m^3^/ha/year) in the two field trials evaluated. There were also no significantly different predicted volume values between the event receiving the sprayed treatment and the event under control conditions. These data demonstrated that there was no interaction between treatments and environmental conditions and evaluation time points in the two field trials. Thus, the tolerance to glyphosate of the tolerance to glyphosate-tolerant eucalyptus events regardless of the planting location or the age of evaluation (Figure 6).

## Discussion

This study describes performance evaluation experiments conducted on the glyphosate-tolerant eucalyptus events 751K022, 955S019, and 955S024, which have been approved for commercial planting in Brazil and have passed extensive biosafety evaluations.^24,37,45^ Glyphosate-tolerant GM crops expressing the CP4-EPSPS protein were first deregulated in the U.S.A. in the late 1990s and rapidly adopted.^37,46–48^ The CP4-EPSPS protein has a long history of safe use in the environment and as food and feed.^37,46,49,50^ Glyphosate is a broad-spectrum herbicide that inhibits the enzyme EPSPS, which is involved in the synthesis of aromatic amino acids (tryptophan, tyrosine, and phenylalanine) in plants.^51,52^ The inhibition of EPSPS disrupts the shikimate pathway, leading to the depletion of essential amino acids and eventual plant death.^39,53,54^ Growth inhibition is the first visible symptom of glyphosate’s phytotoxic effect.^43,55^

A drastic reduction in growth parameters was observed in conventional clones FGN-K and FGN-S treated with commercial sub-doses of glyphosate. The dose of 0.45 kg a.e. ha^−1^ (25% of the commercial dose) caused a 52% reduction in biomass accumulation for WT FGN-K and WT FGN-S (Figure 2a). These results align with previous study that showed a 52–56% reduction in dry biomass in *Eucalyptus grandis* x *Eucalyptus urophylla* hybrids 30 days after the spray application of 0.45 kg a.e. ha^−1^ glyphosate.^43^ These results highlight the high susceptibility of eucalyptus seedlings to glyphosate, indicating that drift onto eucalyptus seedlings can severely compromise initial stand development.^25,28^

During the development of herbicide-tolerant events for various species, several criteria must be met to select events for deregulation and commercial release. The main evaluation criterion is maintained productivity under herbicide application.^56–59^ The event must not suffer from phytotoxicity effects that could reduce its productivity. The glyphosate-tolerant eucalyptus events evaluated in this study went through phenotypic assessments at the seedling stage, photosynthesis testing, and long-term field performance analyses.

Photosynthetic evaluations of carbon assimilation showed that glyphosate-tolerant events maintained their photosynthetic efficiency shortly after glyphosate application. The CO_2_ flux (AQY) values in treated WT events began to decline five days after application. For example, event 751K022 presented an average AQY of 0.077 μmol m^−2^ s^−1^ at 5 DAA, while WT FGN-K presented an average AQY of 0.058, a 24.6% reduction in CO^2^ flux. FGN-S showed 16% and 11.2% reductions in AQY compared to 955S019 and 955S024, respectively. Fv/Fm (maximum quantum efficiency) reductions between 4% and 33% were reported for three eucalyptus clones seven days after the application of 0.18 kg a.e. ha glyphosate.^43^ These reductions are directly related to reduced photosynthetic capacity and increased radiation requirements for CO^2^ fixation, subsequently reducing Amax and AQY.^60–62^ This study shows that glyphosate significantly affects biochemical reactions of photosynthesis in WT clones, while the GM events maintain photosynthetic capacity even when exposed to high levels of glyphosate.

Long-term productivity assessments in the field showed that early over-the-top applications of glyphosate did not influence the performance of the glyphosate-tolerant eucalyptus events, with productivity statistically similar to non-sprayed WTs. Although the potential drift in WT clones and operational gains with mechanized over-the-top glyphosate application in glyphosate-resistant events were not estimated, the introduction of glyphosate-tolerant eucalyptus into integrated weed management holds great potential for enhancing eucalyptus farm productivity.

In soybean, photosynthetic parameters of both first (RR) and second (RR2) generation glyphosate-tolerant cultivars were negatively affected by increased glyphosate doses and late applications, resulting in decreased leaf area, shoot biomass production, and yield.^63–66^ In contrast, low doses and early applications caused less damage, suggesting that early applications allow more time for plants to recover from glyphosate or its metabolite effects. In eucalyptus, glyphosate applications are concentrated in the first 90 days after planting, generally performed at 30-day intervals. This contrasts with annual crops like soybean, corn, and cotton, whose production cycles often take between 90 and 150 days. Although weed competition control is concentrated in the first year of the eucalyptus seven-year cycle, studies have demonstrated that weed competition and herbicide drift can cause long-term losses of up to 90% in potential productivity.^13,20,26,28,67,68^

Varied eucalyptus responses to low glyphosate doses have been reported.^25,28,69^ However, at commercial doses, all eucalyptus species were sensitive,^43^ demonstrating no genetic variability for glyphosate tolerance and the need to breed the trait into the population. Although the transformed eucalyptus events (T_0_ generation) may be useful in operational plantations, they are valuable for breeding as a complementary resource for traditional tree breeding programs.^70^ The introgression of the glyphosate tolerance trait should be carried out by crossing GM events with parents with high combining capacity, enabling selection in the first generation of GM derivatives (T_1_) for clones with suitable quantitative traits for different planting environments and high glyphosate tolerance.

A broad-spectrum herbicide as glyphosate is frequently used to eradicate weeds and other undesirable plants.^48,71^ However, glyphosate as a sole herbicide has created strong selection pressure for weeds that have, through natural variation, developed resistance mechanisms.^72^ There are 48 weed species that have evolved glyphosate resistance, increased over the years in the agricultural sector.^73,74^ In Eucalyptus cultivation, the migration of glyphosate-resistant species is primarily linked to regions with extensive agricultural practices that involve frequent glyphosate application.^74^ This consistent use can lead to the selection of resistant weed populations.

In weed control management in eucalyptus, glyphosate is typically applied only two to three times within the first four months after planting, followed by a significant gap of around six years without herbicide intervention.^13,15,75^ Thus, to better understand the implications of using glyphosate-tolerant eucalyptus, further studies should focus on the role of glyphosate in integrated weed management, emphasizing not just the strategic use of glyphosate, but also the incorporation of diverse herbicide molecules. This approach could help mitigate resistance development and enhance overall weed control efficacy in eucalyptus stands.

In this study, early evaluation in seedlings, photosynthetic-physiological assessments, and long-term field performance demonstrated high glyphosate tolerance in events 751K022, 955S019, and 955S024. All events exhibited growth patterns, biomass, and overall photosynthetic parameters comparable to their wild-type counterparts, even under increasing doses of glyphosate exposure. These results represent a significant advancement in cp4-epsps gene utility, previously demonstrated in several other crop species, resulting in improved weed control efficacy with clear benefits in terms of sustainability.^71,76^ Introducing these glyphosate-tolerant eucalyptus events can provide sustainable, environmentally friendly, targeted, and effective weed control by reducing weed competition and preventing unintended drift damage in young stands. Furthermore, these expected advantages of using glyphosate-tolerant eucalyptus should be evaluated in a large-scale field trial with the events characterized here in this study as well as combining glyphosate tolerance with tolerance to other herbicides through stacking should enable more diverse management, integrating approaches for sustainable weed control.

## Data Availability

The data presented in this study are available on request from the corresponding author.
